# A systematic review of laser therapy for vulvovaginal atrophy/genitourinary syndrome of menopause in breast cancer survivors

**DOI:** 10.3332/ecancer.2019.988

**Published:** 2019-12-12

**Authors:** Charity Knight, Vera Logan, Deborah Fenlon

**Affiliations:** 1 Department of Obstetrics and Gynaecology, Singleton Hospital, Swansea SA2 8PP, UK; 2 Department of Nursing, College of Human and Health Sciences, Swansea University, Swansea SA2 8PP, UK

**Keywords:** atrophic vaginitis, breast neoplasms, laser therapy, cancer survivors

## Abstract

Women who have been treated for breast cancer may experience vulvo-vaginal atrophy (VVA)/genitourinary syndrome of menopause (GSM). This is a progressive condition and will not improve without treatment. Whilst vaginal oestrogen is the most effective treatment for GSM, many breast cancer survivors and clinicians remain reluctant to use it. Laser therapy is emerging as an alternative treatment for this condition but there is little evidence available as to its value in this setting.

We undertook a systematic literature review to identify available evidence for the use of laser therapy for VVA in women with breast cancer. There are a number of small studies which suggest an improvement in vaginal health in this group. However, these are all small, non-randomised studies and there are a number of key questions which need to be answered before this treatment can be implemented into practice.

## Background

Vulvovaginal atrophy (VVA), which is now known as genitourinary syndrome of menopause (GSM), results due to the natural hypo-oestrogenic state that occurs during and following menopause. Approximately 50% of post-menopausal women will suffer from GSM (reported incidence is between 39% and 62%) [20]. However, up to 75% of women surviving breast cancer are expected to experience GSM [27], because they are either post-menopausal at diagnosis or they have become menopausal as a result of endocrine or chemotherapy. Achieving very low oestrogen levels is the goal of endocrine sensitive breast cancer treatment, which will exacerbate menopausal problems. It is estimated that there are over 500,000 breast cancer survivors living in the UK, with the majority of women surviving for over 20 years. GSM is progressive in nature; hence, it will only deteriorate with time and will not improve without intervention. This can have a significant effect on the quality of life (QoL) [7].

The vaginal mucosa prior to menopause is composed of a thick stratified squamous epithelium [8] which is hormone-responsive, relying primarily on oestrogen to maintain epithelial turn-over. Oestrogen receptors have been identified throughout the urogenital tract, and also in the vulva, vagina, urethra and trigone of the bladder [11]. Epithelial exfoliation leads to a release of glycogen which fuels *Lactobacilli vaginalis*, producing lactic acid to maintain a low vaginal pH. This inhibits the colonization of pathogenic bacteria and fungi [28]. With the onset of menopause or hypo-oestrogenic state, the vaginal mucosa thins out, leading to a dramatic reduction in epithelial exfoliation into the vagina. This leads to a cascade of events, resulting in a reduced *Lactobacilli* commensal population, a higher vaginal pH and an increased presence of pathogens, hence creating an increased risk of vaginal infection [11] and GSM symptoms.

GSM is significantly under-reported by women, who are reluctant to volunteer symptoms, with less than 25% of women seeking medical treatment [20] and it is poorly recognized by clinicians when conducting a gynaecological review. GSM presents with a broad spectrum of symptoms which can be divided into three symptom types: vulvovaginal discomfort, sexual dysfunction and urinary symptoms. As many as 45% of post-menopausal women experience vaginal symptoms [25]. Specific symptoms of GSM reported in the women’s EMPOWER survey [9] were: vaginal dryness (79%), itch/irritation (77%) and dyspareunia (59%). The VIVA study [14] reported dryness (83%), burning (14%), pain with touch (11%) and urinary incontinence (30%) with 62% describing the severity of these symptoms as moderate or severe [14]. Apart from physical symptoms, many women report that vaginal atrophy has a negative impact on their quality of life, sex, relationship and self-esteem [14]. Symptom levels may be worse in women who do not currently or have never taken hormonal treatment, with GSM symptoms reported in 60% of cases and over 90% experiencing these as bothersome [25].

Management for GSM symptoms includes topical treatment with vaginal moisturizers, lubricants, systemic and/or topical oestrogen therapy [2]. Specific treatments for the management of sexual dysfunction include the use of vaginal lubricants and dilators, local anaesthetics (topical/injected), physiotherapy and psychosexual counselling [12]. Specific treatment of urinary symptoms includes lifestyle modifications, physiotherapy, oral medication and vaginal oestrogen [16]. A recent Cochrane review suggests that vaginal oestrogen preparations improve GSM symptoms with no difference comparing the different vaginal oestrogen products (oestrogen cream, tablets and ring) [10]. Vaginal oestrogen has also been shown to be effective in reducing the incidence of recurrent UTI and may even be more effective than antibiotic treatment [21].

While vaginal moisturisers and lubricants provide topical relief for GSM, their effect is short-lasting (Edwards and Panay, 2016), and it is widely recognised that systemic and/or topical oestrogen provides the most effective treatment [17]. However, systemic hormone replacement therapy (HRT) is contraindicated in those with a history of breast cancer [23]. GSM is particularly bothersome in breast cancer survivors as there is often both clinician and patient reluctance to use topical oestrogen treatment [2]. The North American Menopause Society (NAMS) recommend an assessment by the oncology team considering risk and benefit on an individual patient basis.

A recent NICE clinical pathway developed for the management of menopause [18] suggests referral to a clinician with menopause expertise. However, most women with breast cancer do not see a gynaecologist and there are very few specialist menopause clinics available nationally. Currently, very few women with breast cancer are offered anything in the way of care or management of this problem.

Laser therapy for the vagina has recently become available in the UK but only in the context of private health care. The use of fractional CO_2_ laser and vaginal erbium:YAG laser (VEL) has been shown to be effective for the treatment of vaginal dryness and dyspareunia and may improve mild-to-moderate stress incontinence and vaginal prolapse [3]. The carbon dioxide (CO_2_) laser was initially introduced in the 1980s for plastic surgery procedures used to implement skin resurfacing for the treatment of scarring, skin damage and ageing effects [6]. Since then, a number of different laser systems have been developed for the treatment of gynaecological conditions and the use of fractional CO_2_ vaginal laser since an alternative treatment for GSM has gradually increased following the seminal study by Salvatore *et al* [24]. This was a pilot study of 50 women with refractory GSM symptoms (following vaginal oestrogen) who received three courses of CO_2_ fractional vaginal laser over 12 weeks. They reported significant improvement (*p* < 0.001) in GSM symptoms and vaginal health index (VHI) score at a 3-month review. The women experienced minimal discomfort with the insertion and movement of the probe, and satisfaction with the laser procedure was reported in 42 (82%) of the women.

Early studies by Gaspar *et al* [5] described histological improvements in the vaginal mucosa following the use of fractional CO_2_ vaginal laser when combined with local platelet-rich plasma (PRP) compared to a control group that received local PRP without vaginal laser. In biopsies taken from women with vaginal atrophy, the treatment group were reported to have an increase in the ‘thickness of the vaginal epithelium’, increased glycogen density and neoangiogenesis, when compared to the control group. A later study by Zerbinati *et al* [28] reports similar findings in symptomatic menopausal women, who received fractional CO_2_ vaginal laser, with a comparison between pre-treatment and post-treatment biopsies. Both light and electron microscopy described a striking improvement in the stratified, squamous epithelium, which increased from a 5–10 cell layer depth to a 20–40 cell layer depth with glycogen-rich, superficial cells shedding from the surface layer and vessel-rich papillae, at 1 month following the treatment, which were maintained at 2 months. This post-treatment histological change was supported by an improvement in VHI score and all clinical symptoms, with a statistically significant improvement in vaginal dryness, burning, itching, dysuria and dyspareunia.

Further work by Sokol and Karram [26] has shown significant improvements to VVA lasting over 1 year with Gaspar *et al* (2017) suggesting that laser treatment may be more effective in the long term than topical oestrogen, as symptoms tend to recur once oestrogen is discontinued. A number of studies have shown that in breast cancer patients, vaginal laser therapy can improve symptoms of VVA; however, there are no large-scale randomised controlled trials of laser therapy in women with breast cancer. It is not known whether this treatment would be acceptable to women and what the benefits might be in the long term. Some clinicians are prepared to prescribe vaginal oestrogen to women with breast cancer, but some women may prefer to have non-hormonal options. It is also not clear how much treatment would be necessary to achieve symptomatic relief in women with breast cancer and how much follow-up would be required. There is an urgent need for studies to begin to address this potentially highly effective treatment to improve the long-term health of women with breast cancer. In order to develop appropriate studies, a literature review has been undertaken to ensure that they are based on the current, most up-to-date knowledge. The aim of the literature review was to explore the current evidence about the benefits of laser therapy in breast cancer survivors with vaginal atrophy.

## Methods

A systematic review was undertaken to ask what is currently known about the benefits of laser therapy in breast cancer survivors with vaginal atrophy.

Further objectives of the review were:

- Are there any patient-reported benefits such as improved dryness, dyspareunia and itching?
- Are there any observed benefits, such as visual improvements in vaginal mucosa (including vaginal colour, epithelial integrity, vaginal epithelial surface thickness and vaginal secretions)?
- What current treatment regimens are used?
- How long does any benefit last?

The selection criteria for the literature was to include primary evidence from trials, randomised or otherwise. Using the PICO framework, the population were females following breast cancer treatment with a diagnosis of GSM or vulvovaginal atrophy. The intervention was laser therapy, either CO_2_ or Er:YAG. The comparative intervention was of any type. Outcomes could include gynaecological, histological or patient-reported outcomes. Studies were identified by computerised searches of the following databases in May 2018: Medline (PubMed), Scopus, Web of Science, Cochrane Library and ClinicalTrials.gov. The following combinations of index terms and free text were used for each search strategy: breast cancer OR breast neoplasm* OR carcinoma, ductal, breast AND laser therapy OR laser* AND vaginal diseases+ OR vaginal atrophy OR vagina OR vagina/PA OR GSM. No limits were used, including no time limit. EU Clinical Trials Register, Clinicaltrials.gov and ANZ clinical trial registry were also searched for randomised controlled trials in progress. Reference lists of eligible articles were hand-searched to identify additional literature.

Included studies assessed the effect of laser therapy on the vaginal symptoms of GSM/VVA in females following breast cancer treatment. Studies were first screened by title and abstract, and then the full text of the selected studies was obtained and reviewed, resulting in a final selection of six studies which m*et all* criteria.

**Figure:**
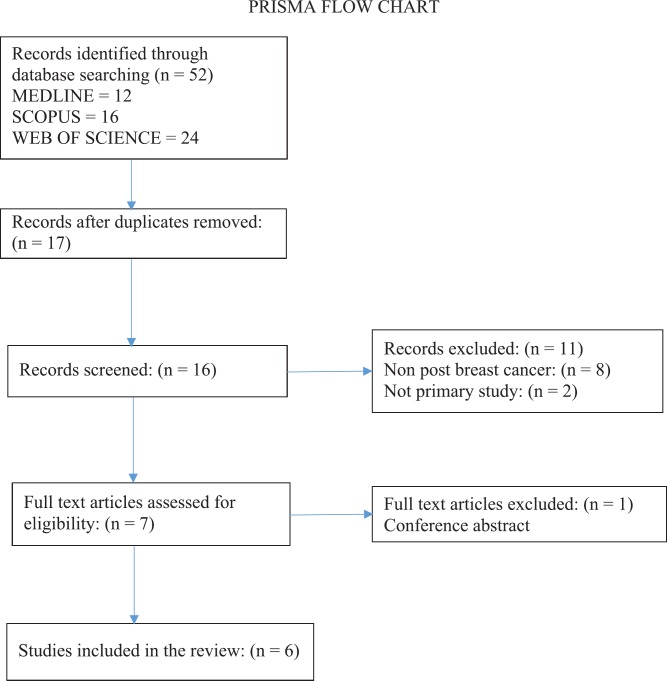

## Results

### Characteristics of the studies

Table 1 summarises the characteristics of articles that met the inclusion criteria.

Six studies were found which met the inclusion criteria, four of which were conducted in Italy, one in Germany and one in the USA. Sample sizes were small in all studies, ranging from 8 to 50 patients. The combined number of women as a total of all the studies presented here is 163. The participant eligibility criteria always included post breast cancer and vaginal/pelvic floor symptoms; however, the type of symptoms and some additional criteria differed between the studies. For example, one study only included women following prolapse surgery (Mothers *et al* 2017). Other studies’ inclusion criteria comprised combinations of GSM symptoms, dyspareunia and vaginal atrophy. This suggests that the aims of the studies were varied, with some focusing on vaginal symptoms, some on dyspareunia and some on pelvic floor symptoms. In the Pieralli *et al* study, 44% of the patients were receiving adjuvant therapy with Aromatose Inhibitors (9%) or Tamoxifen (91%). None of the studies were randomised and two were retrospective reviews (Mothers *et al* 2017, Pagano *et al* 2016).

### Characteristics of the interventions

The interventions used varied regarding the type of laser used: four of the studies examined the effect of CO_2_ laser and two investigated the effect of Er:YAG laser. Further differences stemmed from treatment regimens, ranging from one to three treatments, length of exposure and some of the laser settings (see Table 2 for the particulars on the type of laser and the therapeutic protocol). It was not clear whether one form of laser treatment had better outcomes than the other.

### Effectiveness of the interventions

Table 3 summarises the outcome measures that improved significantly.

The effectiveness of interventions was measured by a combination of subjective and objective measures. All studies reported statistically significant benefits of laser therapy. The four studies that evaluated (VHI) vaginal health index found significant improvements in this measure. Subjective evaluations of symptom relief or condition improvement, examined in all studies through methods of VAS, VRS, EGGS, FSFI, WBFS, FSDSr or combination of the above, also showed significant positive changes after treatment.

Four of the studies used CO2 laser with the following improvement of the GSM clinical symptoms. Becorpi *et al* (2017) reported significant changes in VRS, FSFI and VHI, with no significant FSDSr change) and significant changes in inflammatory and modulatory cytokine patterns, without modifications in the vaginal microbiome after the treatment. Gittens *et al* (2018) noted significant improvement in FSFI, WBFS for pain, dyspareunia and vaginal dryness and FSDSr. Pagano *et al* (2016) reported significant VAS score improvement in sensitivity during sexual intercourse, vaginal dryness, itching/stinging, dyspareunia, dysuria and bleeding. Pieralli *et al* (2016) noted significant improvement in VHI scores.

Of the two studies that used Erbium: YAG laser, Gambacciani *et al* (2017) reported significant improvement in vaginal dryness and dyspareunia VAS scores, as well as VHIS. Mothers *et al* (2017) reported significant positive change in VHI, non-significant change in pH from 5 to 4.8 (p=0.14) and 94% overall satisfaction with symptom relief.

Although significant benefits were reported, most of the studies only evaluated the effects of treatment in the short term (one or two months post intervention), with only two papers looking at both, short- and long-term evaluation. Gambacciani *et al* (2017) found significant changes (objective and subjective) lasting for 12 months. There was a trend for maintained improvement up to 18 months, but this was not significant. Pierrali *et al* (2016) conducted a telephone satisfaction survey varying from three to 25 months after treatment. They reported that 52% of patients were either satisfied or very satisfied with the treatment results. This was a drop from 76% reporting either satisfied or very satisfied immediately after the treatment, which suggests that the effects may diminish over time. No severe adverse effects were reported.

## Discussion

Although all the studies in this review were small and heterogenous in terms of the type of laser used and the outcomes assessed, they all showed a rapid and statistically significant benefit for laser treatment on vaginal tissues, with significant improvements in both self-reported and observed measures lasting up to 12 months. Most studies did not evaluate the long-term effects of the treatment, with the exception of Gambacciani *et al* (2017), who found that statistically non-significant improvements lasted past 18 months after the treatment. Further work is required to know whether changes are long term and whether retreatment is required, as well as how often treatment should be repeated.

This review gives no indication as to which type of laser treatment was either more effective, safe or better tolerated. Both the fractional microablative CO_2_ lasers and the non-ablative photothermal Er:YAG lasers have been used in the studies. Comparison is further complicated by differing durations, number of treatments and variances in reporting of laser settings, with a further option of manipulating the frequency and intensity. While both lasers generated improvements in GSM symptoms, the exact mechanism of action remains unclear. To help clinicians and women suffering from GSM make an informed decision as to which type of laser and what therapeutic protocol should be used, good quality trials are needed by comparing the options.

The outcome measures in these studies varied. Self-reported measures were usually asked either on a scale of 1–10, or 1–5, on a variety of symptoms, including vaginal dryness, dyspareunia, itching, burning or sting, reduced sensitivity during intercourse, dysuria, vaginal bleeding and leucorrhoea, or general satisfaction with symptom relief. However, these were not standardised and it was not clear how they were asked or recorded by the patient or the clinician; these were the most important issues for the women experiencing these symptoms. Two validated measures were used by Becorpi *et al* [1] to measure sexual functioning: the FSFI = female sexual function index and the FSDSr = female sexual distress scale. The VHI was reported by most studies. Although this is rated by an external observer, this is still subject to inter-observer variation and bias. There is a need for a validated scale to explore and measure those issues which are of specific distress to women experiencing VVA so that comparisons can be made across studies.

This review has several limitations. Despite no applied restrictions, only six studies of moderate or poor quality were identified. None of the studies were randomised, either to another treatment or to standard care, hence the influence of placebo cannot be ruled out. Two of the studies were retrospective. The heterogeneity of the type of laser implemented and outcome measures make the study unsuitable for meta-analysis. There is also a possible observer and publication bias.

## Conclusion

There are a number of small-scale studies which all suggest an improvement in vaginal health in women who have had breast cancer, both objectively and subjectively. However, there are no large-scale studies which discuss the acceptability of the intervention and which were randomised. There are a number of questions to be asked before this intervention can be widely accepted into clinical practice, including which kind of laser therapy, how many treatments are required, what is most acceptable to patients, how often treatment needs to be repeated, what kind of benefit is obtained and whether this intervention shows significant health benefits over alternatives. There is, therefore, a need to undertake large-scale, prospective, randomised controlled trials to fully explore the benefits of vaginal laser as a therapy for vaginal atrophy and to gain a better understanding of whether this treatment can reduce symptom burden and improve QoL for post-menopausal women, particularly after breast cancer treatment.

## Conflicts of interest

No authors have any competing conflicts of interest.

## Figures and Tables

**Table 1.** Study details and demographics.

First author	Country	Aims of the study	Study design	Sample size	Demographics and clinical details	Inclusion criteria
Becorpi *et al* [1]	Italy	To evaluate the effects of laser treatment on vaginal immune mediators and microbiome.	Prospective, non-randomised, uncontrolled.	20	mean age: 58.2 years, mean BMI: 23.7 kg/m^2^, mean age of menopause: 12.4 years, post menopause: 8.85 years.	Menopausal status and diagnosis of vaginal atrophy and previous breast CA.
Gambacciani (2017)	Italy	To evaluate the short-term efficacy and acceptability of a second-generation vaginal laser treatment for the management of GSM.	Prospective, non-randomised, uncontrolled.	43	mean age = 50.8 yrs (range: 38–70), age at menopause = 43.2 years (range: 31–55), post menopause: 9 years (range: 1–18).	Post-menopausal, breast CA survivors, suffering from GSM.
Gittens (2018)	USA	To examine the outcomes of sexual function in women with GSM symptoms post endocrine therapy for breast CA.	Prospective, non-randomised, uncontrolled.	8	Mean age = 55.2, mean age of menopause: 47.3, average duration of symptoms = 9.4 years, all treated with endocrine therapy, four patients still being treated during the course of the study.	Post-menopausal, breast cancer survivals, symptoms of GSM.
Mothes *et al* [13]	Germany	To evaluate the efficacy of dual-phase Er:YAG technology in atrophy-related urogynecological symptoms after prolapse surgery in patients after breast CA.	Retrospective, non-randomised, uncontrolled.	16	age: 71 years (SD = 7).	Breast CA survivors, following prolapse surgery, with pelvic floor symptoms related to vaginal atrophy.
Pagano (2016)	Italy	To evaluate the efficacy and safety of the CO_2_ laser technique for women with VVA.	Retrospective, non-randomised, uncontrolled.	26	age: 20–62 years, (median = 42) 1 woman was post-menopausal before starting chemo /hormonal therapy + received adjuvant antioestrogen therapy, 25 women had therapy-related menopause, 22 women: adjuvant chemotherapy.	Hormone-receptor positive breast cancer, VVA symptoms, post surgery.
Pieralli *et al* [22]	Italy	To assess the efficacy of fractional CO_2_ laser therapy for VVA dyspareunia.	Prospective, non-randomised, uncontrolled.	50	Mean age 53.3 years (range 41–66) no adjuvant therapy *n* = 28 adjuvant tamoxifen *n* = 20, adjuvant aromatase inhibitors *n* = 2. VHI evaluated in first 36 patients only.	Current or previous breast CA, Oncological menopause (mean time of menopause = 6.6 years, range 1–17) VVA, dyspareunia.

**Table 2.** Type of laser, therapeutic protocol and results.

First author	Type of laser	Therapeutic protocol	Follow-up interval	Outcome measures	Results	Comments
Becorpi *et al* [1]	CO_2_	Two treatments, power 30 W, dwell time 1000, dot spacing 1000, 2 shots, 45°, total exposure: 3–5 min.	1 Month	Primary: VRS* (signs of VVA) VHI* FSFI* FSDSr* Secondary: vaginal cytokines.	Significant reduction of clinical symptoms (VRS) (*p* range: 0.000–0.012), FSFI *(p* = 0.003) and VHI (*p* = 0.000) scores, apart from dysuria values (*p* = 0.132). Non-significant changes in FSDSr (*p* =0.074), and vaginal microbiome (*p* = 0.7), mostly significant changes in inflammatory and modulatory cytokine patterns (*p* range: 0.000–0.970).	No long-term evaluation of effects, small participant number, single centre, VHI scores significantly lower in comparison with other studies.
Gambacciani (2017)	Er:YAG	Three treatments, wavelength 2940 nm, dia of spot 7 mm, pulse freq: 1.6 Hz, fluence 6 J/cm^2^ (3 shots vag wall, then vestibule and introitus).	1 Month, 3 months, 6 months, 12 months and 18 months	– VAS* (vag. dryness and dyspareunia) – VHI*	Significant improvement in symptoms of vaginal dryness (*p* < 0.01 versus basal values) and dyspareunia (*p* < 0.01 versus basal values), as well as VHIS scores (*p* < 0.01 versus basal values). Non-significant changes in all measured scores after 18 months post treatment (NS versus basal values).	Pilot study, single centre.
Gittens (2018)	CO_2_	Three treatments, laser settings not disclosed.	6 Weeks	FSFI* WBFS* (pain, dyspareunia, vaginal itching/burning/dryness, dysuria) FSDSr.	Significant improvement in FSFI (*p* = 0.044 or less), WBFS (*p* = 0.066 or less, apart from dysuria: *p* = 0.351) and FSDSr (*p* = 0.002).	Small sample size, lack of long-term follow-up.
Mothes et al [13]	Er:YAG	One treatment, 2940 nm, Dual phase: fractional mode (300 ns pulse duration) Thermal (1000 ns) 10 min.	2 Months	– EGGS* (symptom relief) – pH – VHI*	Significant improvement in VHI scores (p = 0.01) no significant changes in pH scores (p = 0.14). 94% positive patient evaluation.	Sample not representative of younger population (following prolapse surgery), different inclusion criteria to other studies (low grade stress urinary incontinence), making comparisons to other studies difficult, small participant number, single centre, missing details on adjuvant cancer therapies, no long-term evaluation of effects.
Pagano (2016)	CO_2_	Three treatments, power 30 W, dwell time 1000 ns, dot spacing 1000 nm, Smart stack parameters 1–3.	1 Month	– VAS* (VVA symptoms, procedure-related discomfort).	Significant regression of VVA symptoms, VAS scores for dyspareunia, dryness, itching/stinging and sensitivity during sexual intercourse were 78%,80%,75% and 86%, respectively, lower than baseline, (*p* < 0.0001) and procedure-related discomfort (*p* < 0.0167).	Subjective outcome measures only, no long-term evaluation of effects, small number of participants, single centre study.
Pieralli *et al* [22]	CO_2_	Three treatments, power 30 W, dwell time 1000 ns, dot spacing 1000 nm, Smart stack parameter 1, 2 single shots at 45°.	1 Month, 11 months	– VHI* – VAS* (intensity of VVA dyspareunia) Likert scale treatment satisfaction.	Improvement in dyspareunia symptoms (*p* < 1.8) and VHI (*p* < 0.0001). 76% of patients were satisfied or very satisfied with the procedure 4 weeks after the last laser application.	Variable long-term follow-up time (3–25 month), variable adjuvant therapy, small participant number, single centre, subjective measure of long-term follow up, only first 36 patients had VHI evaluated.

* VRS, Verbal rating scale; VAS, visual analog score; VHI, vaginal health index score (elasticity, fluid volume, pH, epithelial integrity and moisture); FSFI, female sexual function index; FSDSr, female sexual distress scale—revised; WBFS, Wong-Baker faces scale; EGGS, goal setting, expectations, goal achievement, satisfaction

**Table 3.** Details of outcome measures that improved significantly (p < 0.01) following laser treatment for VVA in women with breast cancer.

First author	Becorpi *et al* [1]		Gambacciani (2017)		Gittens (2018)		Mothes *et al* [13]		Pagano (2016)		Pieralli *et al* [22]		
Follow-up interval	1 Month		1, 3, 6, 12 Months		6 Weeks		2 Months		1 Month		1 Month, 11 Months		
VAS dryness	Baseline: median [25th–75th percentile range].	After treatment: median [25th–75th percentile range].	Months post treatment	Improvement	N/A		N/A		Baseline: median (range)	After third treatment: median (range)	N/A		
			1	4.7±1.2 cm									
	2 [2–3]	2 [1–2]											
			3	3.8±1.5 cm									
									10 (7–10)	2 (0–5)			
			6	4.9±1.5 cm									
			12	5.5±1.5 cm									
									80% lower than baseline				
VAS dyspareunia	Baseline: median [25th–75th percentile range]	After treatment: median [25th–75th percentile range]	Months post treatment	Improvement	N/A		N/A		Baseline: median (range)	After third treatment: median (range)	Basline: median (range)	After treatment: median (range)	
									9 (5–10)	2 (0–5)	5 (1–5)	3 (1–5)	
	2 [2–3]	2 [1–2]	1	4.2±1.5 cm									
									78% lower than baseline				
			3	4.3±1.8 cm									
			6	4.8±1.9 cm									
			12	5.1±1.8 cm									
VAS itching/stinging	Baseline: median [25th–75th percentile range]	After treatment: median [25th–75th percentile range].	N/A		N/A		N/A		Baseline: median (range)	After third treatment: median (range)	N/A		
	1[0–2.75]	0 [0–1]							8 (2–10)	2 (0–6)			
									75% lower than baseline				
VAS reduced sensitivity during sexual intercourse	N/A		N/A		N/A		N/A		Baseline: median (range)	After third treatment: median (range)	N/A		
									7 (0–10)	1 (0–5)			
									86% lower than baseline				
VAS dysuria, vaginal bleeding and leucorrhoea	Baseline: median [25th–75th percentile range]	After treatment: median [25th–75th percentile range]	N/A		N/A		N/A		Baselinemedian (range)	After third treatment: median (range)	N/A		
	0[0–1.75]	0 [0–0.75]							5 (0–10)	0 (0-6)			
									100% lower than baseline				
VHI	Baseline: median [25th–75th percentile range]	After treatment: median [25th–75th percentile range]	Months post treatment	Improvement	N/A		Baseline:	After treatment:	N/A		Baseline:		After treatment:
							16 (SD 4.6) pH = 5	20 (SD 3) pH = 4.8					
	12 [11–13]	16 [15.25–18]	1	20.0±1.0									
											8.9 (SD 1.7)		21.6 (SD 1.6)
			3	21.0±1.4									
			6	19.7±1.4									
			12	18±1.8									
FSFI	Baseline: median [25th–75th percentile range]	After treatment: median [25th–75th percentile range]	N/A		Baseline to third treatment improvement: 12.48±7.70.		N/A		N/A		N/A		
	27.5 [4–54.50]	43 [20.25–70.50]											
VRS – Symptoms:	Vaginal dryness, dyspareunia, vaginal itching and burning, flattening of vaginal folds, vaginal mucosa dryness vaginal pallor, fragility of the mucosa, petechiae.		N/A		N/A		N/A		N/A		N/A		
EGGS	N/A		N/A		N/A		94% patients satisfied with symptom relief.		N/A		N/A		
WBFS	N/A		N/A		Symptom	Improvement	N/A		N/A		N/A		
					Pain	4.14±2.67							
					Dyspareunia	4.25±3.45							
FSDSr	N/A		N/A		Sexual distress improvement: 18.7±9.25		N/A		N/A		N/A		

*Using Mood’s median test
